# A Review of Parameter Settings for Invasive and Non-invasive Vagus Nerve Stimulation (VNS) Applied in Neurological and Psychiatric Disorders

**DOI:** 10.3389/fnins.2021.709436

**Published:** 2021-07-13

**Authors:** Sean L. Thompson, Georgia H. O’Leary, Christopher W. Austelle, Elise Gruber, Alex T. Kahn, Andrew J. Manett, Baron Short, Bashar W. Badran

**Affiliations:** Department of Psychiatry and Behavioral Sciences, Medical University of South Carolina, Charleston, SC, United States

**Keywords:** VNS, taVNS, tVNS, parameter optimization, neuroplasticity, rehabilitation, epilepsy, depression

## Abstract

Vagus nerve stimulation (VNS) is an established form of neuromodulation with a long history of promising applications. Earliest reports of VNS in the literature date to the late 1800’s in experiments conducted by Dr. James Corning. Over the past century, both invasive and non-invasive VNS have demonstrated promise in treating a variety of disorders, including epilepsy, depression, and post-stroke motor rehabilitation. As VNS continues to rapidly grow in popularity and application, the field generally lacks a consensus on optimum stimulation parameters. Stimulation parameters have a significant impact on the efficacy of neuromodulation, and here we will describe the longitudinal evolution of VNS parameters in the following categorical progression: (1) animal models, (2) epilepsy, (3) treatment resistant depression, (4) neuroplasticity and rehabilitation, and (5) transcutaneous auricular VNS (taVNS). We additionally offer a historical perspective of the various applications and summarize the range and most commonly used parameters in over 130 implanted and non-invasive VNS studies over five applications.

## Introduction

The earliest description of electrical stimulation of the vagus nerve began in the 1880’s in New York. Dr. James Corning applied an electric current as an adjunct to his carotid compression fork; other adjuncts included a neck belt and a lower body vacuum chamber. His cases were anecdotal with limited records of the parameters used. Corning argued his device could prevent or terminate seizures by physically compressing blood flow and modifying parasympathetic tone (Lanska, 2002). Since then, researchers have been seeking to refine and optimize vagus nerve stimulation VNS parameters to treat a variety of neuropsychiatric and medical disorders. Stimulation parameters have a significant bearing on the efficacy of neuromodulation, and here we will describe the longitudinal evolution of VNS parameters in the following categorical progression: (1) animal models, (2) epilepsy, (3) treatment resistant depression, (4) neuroplasticity and rehabilitation, and (5) auricular VNS. Each section summarizes the range and most commonly used parameters, while the body of text describes individual studies in a historical narrative. Tables are included summarizing the parameters at the completion of each section.

When discussing parameters of VNS, we most commonly refer to parametric factors that affect the administration and delivery of any electrical stimuli. These influence effective dosage. We establish the important terms throughout the review here:

(a) *Pulse width* is the length of time of a square pulse of current. This time parameter is in microsecond (μs) unit.
(b) *Current intensity* is a measure of the amplitude, or strength, of the electrical pulse. This is in milliampere (mA) unit. Current intensity is a specific parameter in constant current (current-controlled) neurostimulation applications, where an electrical pulse generator varies voltage based on resistance of tissue to maintain stable current intensity. VNS is most often delivered as current-controlled. Current-controlled stimulation has several advantages over voltage-controlled stimulation, including safety and precision control of stimulation. Although VNS may theoretically be administered using voltage-controlled stimulators, current-controlled is the standard for this application.
(c) *Frequency* is a measure of total period cycles (the start of a pulse to the start of the next pulse) in a second. Unlike pulse width, it considers the time with no applied current. This is in hertz (Hz).
(d) *On-Off Time* is the amount of time stimulation and non-stimulation epochs are delivered for during a specific period. The “ON” period is the time that stimulation is delivered above an intensity of 0 mA. The “OFF” period is where no stimulation is delivered (0 mA). In practice, this establishes periods of active stimulation interspersed with periods of rest. If ON/OFF periodic rhythms are delivered as part of intervention, these periods are often repeated for the duration of the intervention.
(e) *Duration* of stimulation is the cumulative time of VNS treatment. For example, a patient receiving daily VNS for 6 months has a duration of 6 months. It is an imprecise measure of dosage because it does not convey how much stimulation is in that time. The significance of duration is that it considers the effect of cumulative dosage.

While many of these parameters have very standard definitions, some of them do not; terms like “duration” are inconsistent across papers to refer to different scales of time. The terms listed above serve simply as an operational platform for discussion here.

**FIGURE 1:**
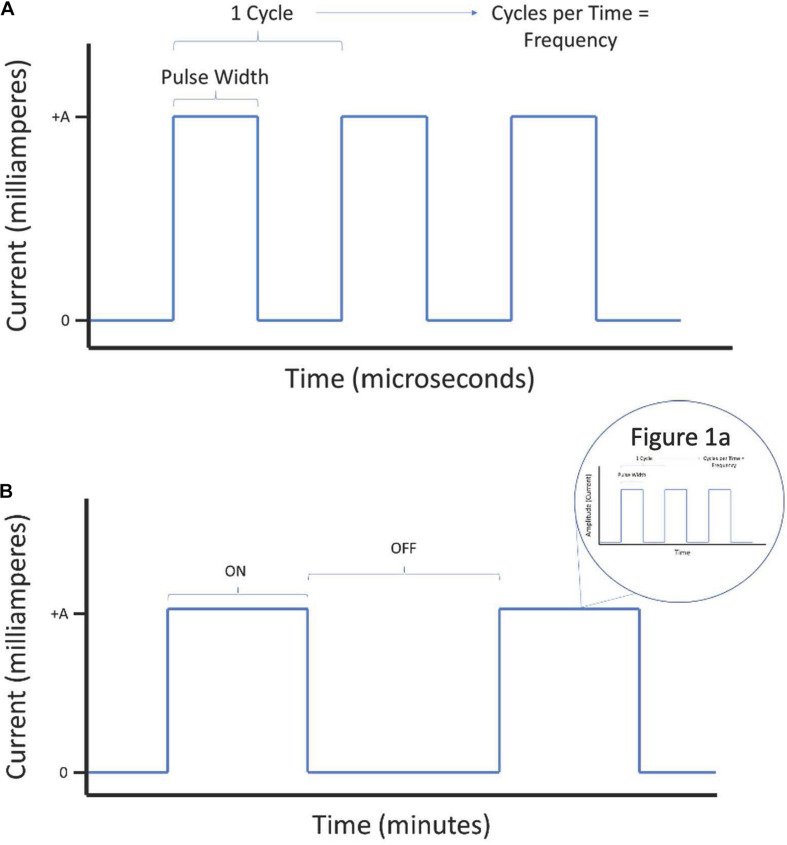
Visual representation of electrical waveform parameters for consideration during administration of vagus nerve stimulation (VNS). The parameters described in the introduction are outlined here on a short time scale **(A)** as well as on a long time scale **(B)**.

Lastly, Figures 1A,B present visual representations of these parameters as simplified electrical waveforms.

## Early Animal Models and Mechanism

### Early Animal Work

Bailey and Bremer (1938) used cats to study the afferent effects of the vagus nerve. This study administered electrical current through nerves proximal to where they severed them and recorded electrograms from the cortex, finding some general activity in the frontal lobe. Regarding parameters, there are still many gaps in this early period; frequency was recorded between 24–50 Hz, and current was “of sufficient strength to evoke a maximal cardiomoderator reflex” (Bailey and Bremer, 1938). Though they tried to control this by creating an “isolated encephalon” model, they did note that blood pressure changes could still affect their measurements. The significance of this in the early work is that it was yet unclear whether VNS had a direct effect on the brain or if it was an effect secondary to peripheral activation.

The next major group known to study VNS in animals was Zanchetti et al. (1952), who used a similar isolated encephalon cat model to Bailey and Bremer. Their findings reveal that VNS could decrease spontaneous cortical spindles and convulsion spindles induced by strychnine. This study used 500 μs pulses from 2–300 Hz; intensity was reported only through the voltage (0.1–2 V) (Zanchetti et al., 1952).

Current scientific standards require more parameters than these measured or reported, so it is hard to draw direct comparisons to later work. However, the work of the aforementioned scientists showed that VNS may act in the CNS (Bailey and Bremer, 1938; Zanchetti et al., 1952; Lanska, 2002).

Further experiments in the 1960s used VNS and EEG to differentiate afferent nerve conduction speeds and subsequent effects (Chase et al., 1967; Chase and Nakamura, 1968). However, this period of VNS research was so parametrically diverse that it is difficult to compare many of these papers. There are wide ranges of parameters even within individual papers, while other parameters are entirely missing until later decades (e.g., current intensity, time administered, and ON-OFF time).

Towards the end of the century, when interest in VNS for epilepsy gained momentum, the core parameters largely stabilized – even in the animal models. For example, Zanchetti et al. (1952) tested across a frequency range of 2–300 Hz; by comparison, almost every animal paper from 1995-onward used either 20 or 30 Hz (Naritoku et al., 1995; Krahl et al., 1998, 2001; Manta et al., 2009; Raedt et al., 2011; Furmaga et al., 2012; Pena et al., 2013; Hays et al., 2014a). Pulse width in animals varied considerably; several papers used 500 μs in the 1990s and early 2000s (Naritoku et al., 1995; Krahl et al., 1998, 2001; Manta et al., 2009). Later pulse width was commonly at 100 μs from around 2010 onward (Hays et al., 2014a,b; Borland et al., 2016; Buell et al., 2019). Current intensity was less consistent, but most papers tended to use less than 1 mA, with 0.8 mA being somewhat more common than others (Hays et al., 2014a; Borland et al., 2019; Buell et al., 2019; Meyers et al., 2019). This was true across studies, many of them for epilepsy but also for other sub-fields including spinal injury, depression, auditory plasticity, and memory; many others simply looked for mechanisms of VNS effects.

### Central Effects of VNS

It is important to consider the afferent, central effects of VNS when discussing potential behavioral effects. Some of the research has looked at the vagus nerve itself, which suggested that small unmyelinated slow-conduction fibers carry the effective signal (Woodbury and Woodbury, 1990; Zabara, 1992). This was challenged by an experiment which selectively lesioned these fibers in rats but did not take away from the anti-epileptic effect (parameters 500 μs, 20 Hz, and 1 mA) (Krahl et al., 2001). Further research would start to shed light on the next steps in this pathway. While there is not a complete model, there is some foundation. Several areas of the nervous system have been proposed, such as the Nucleus Tractus Solitarius and Reticular Formation, as well as more general GABAergic systems, that might be behind the anti-epileptic effects (Woodbury and Woodbury, 1990). C-fos immunostaining in rats showed a broader idea of afferent areas; the parameters used (500 μs pulses, 30 Hz, 30 s ON/5 min OFF, 3 h duration, and 1 mA) resembled those used in human treatments. Using those parameters, the researchers found activations in vagus nuclei, the solitary nucleus, the locus coeruleus, cochlear nucleus, posterior amygdaloid nucleus, cingulate cortex, retrosplenial cortex, hypothalamic nuclei, and the habenular nucleus of the thalamus. They speculated that the limbic system and related areas might account for treating limbic seizures. They also speculated that the noradrenergic locus coeruleus and the solitary nucleus that connects to it might have anti-seizure activity as well (Naritoku et al., 1995).

Research further tested the structure models using similar settings (500 μs, 20 Hz, 30 s ON, and 0.8 mA) with lesions to the LC. In these rats, VNS did not have an antiepileptic effect. This strongly supports the essential role of the noradrenergic LC (Krahl et al., 1998). Using similar parameters, other lesion studies replicated the LC effect and found similar effects with the serotonergic dorsal raphe (500 μs, 20 Hz, 30 s/5 min, and 0.25 mA) (Manta et al., 2009). Blocking alpha-2 signaling with higher frequency (30 Hz) and current (1 mA) and shorter pulse width (250 μs) decreased VNS effects on hippocampal noradrenaline (Raedt et al., 2011). LC lesions also negated antidepressant effects of VNS (250 μs, 30 Hz, and 0.2–0.7 mA) (Grimonprez et al., 2015). Both studies used ON/OFF times of 7 s/18 s, which is a shorter ON time but higher duty cycle than the commonly used 30 s/5 min. Another study depleted norepinephrine and serotonin with immunotoxins and found that they were also necessary for VNS in motor plasticity; the parameters used (100 μs, 30 Hz, 500 m s ON train, 1 week of treatment, and 0.8 mA current) have important differences compared to many epilepsy experiments, so it is promising to see similar neurotransmitters across applications (Hulsey et al., 2019). Acetylcholine depletion further could decrease VNS-paired motor plasticity using parameters similar to Hulsey et al. (2019); Meyers et al. (2019).

Parametric optimization can take advantage of the established neurocircuitry involved to begin to uncover best parameter combinations. Norepinephrine release has been reliably demonstrated to be increased by VNS (12, 23). Hulsey et al. (2017) demonstrated that this is not as straightforward as believed, as higher pulse width and amplitude increase LC firing rate, however, modulating frequency only impacts timing (not firing rate). Current intensity is also shown to be an important parameter as increasing the intensity increases norepinephrine in the cortex and hippocampus (Roosevelt et al., 2006). It is important, however, to understand that more is not always better, as Borland et al. (2016) demonstrated lower neuroplastic effects at the cortex as a function of increased current intensity suggesting a non-monotonic relationship. Activity within the LC and concentrations of neurotransmitters serve as a strong foundation in optimization of VNS parameters.

Animal models have laid the foundation of VNS in almost every application. There are several other studies examined in this section and in Table 1 that are not discussed in detail (Farrand et al., 2017, 2019; Stakenborg et al., 2017; Huffman et al., 2019).

**TABLE 1:** Animal Models (Summary parameters of 36 studies).

	Pulse Width	Frequency	On/Off time	Time administered	Current
Most common Parameter	100 μs (18 uses)	30 Hz (20 uses)	500 m s ON (15 uses)	5 w (3 uses)	0.8 mA (18 uses)
Range of Parameters	100 μs – 4 m s	2–300 Hz	125 m sec – 30 min ON/17.5 s – 5 min OFF	30 s – 6 w	0.2–10 mA

## VNS for Epilepsy

### Epilepsy Animal Models

The Corning Fork of a prior century, initially thought to reduce seizure frequency and long out of use, had a modern successor. Many of the early animal papers focused on EEG and generally revolved around the question: does peripheral vagus stimulation activate the CNS and impact seizures? In the mid-1980s, Zabara (1985 and 1992) used strychnine or PTZ in dogs as a model of seizure. He found that VNS could not only terminate a seizure but could prevent seizures even for some time after VNS stopped. As seen in previous research, cutting the vagus distally did not prevent the effect. Zabara tested a range of pulse widths, frequencies, and current intensities, and suggested that the optimal parameters were ∼200 μs, 20–30 Hz, and 4–20 mA, respectively (Zabara, 1985, 1992). Lockard et al. (1990) similarly, studied VNS moderating provoked seizures in monkeys. Their results were similar, and they studied wide ranges of parameters in their pilot and replication studies. Pulse width was 500–600 μs; currents were 3, 5, or 7 mA; there were many frequencies between 80 and 250 Hz. Where Zabara (1992) used a 30 s ON time, Lockard et al. (1990) stimulated for the duration of a seizure episode or for 40 s after an hour of no seizure activity (Lockard et al., 1990).

There are a few points to make about the canine and primate studies in the context of the larger body of animal VNS work, mostly conducted in rodents. Canine and primate studies used current intensities higher than 1 mA, which is higher than what most research would use for both rat and human VNS for epilepsy. On the other hand, Zabara’s optimized frequency in dogs was similar to what would be used in rats and humans; 20–30 Hz is also regularly used in VNS outside of epilepsy (Zabara, 1985, 1992; Lockard et al., 1990). For comparison, a rat study at that time had similar anti-seizure results but concluded with different optimal parameters: 500–1000 μs, 10–20 Hz, 60 s ON time, and 0.2–0.5 mA per mm^2^ of nerve cross-section (Woodbury and Woodbury, 1990). Another group used rat models at 500 μs and 20 Hz (Krahl et al., 1998, 2001). We can compare VNS studies between species, but it is important to consider that it seems different research groups have settled on different optimal levels even within species.

### Human Epilepsy Trials

Early research on VNS by Zabara had strongly suggested that it would be an effective treatment for seizures, but work remained to show the anti-epileptic effects in humans. Early published data comes from a preliminary paper by Penry and Dean (1990). They tested a range of parameters adapted from the animal models: 130 or 250 μs pulses, 40/47/50 Hz, 29 or 57 s ON, 5 or 10 min OFF, 20 weeks duration (with no stim weeks 8–12), and 1–3 mA as tolerated by their four patients. They saw some reduction in seizure frequencies in three of the patients (Penry and Dean, 1990).

Uthman et al. (1993) used slightly different VNS parameters (500 μs pulses, 50 Hz, up to 120 s ON, 5–20 min OFF, 20 weeks duration with no stimulation weeks 8–12, and current increased as tolerated from 1 mA) in fourteen patients and decreased average seizure frequency by over 45%. They used similar treatment duration and intensity but longer pulse widths and ON periods within each stimulus (Uthman et al., 1993).

Soon after, Wilder et al. (1991) set up a similar study using more patients over at least 24 weeks duration. In this trial, there was a range of initial parameters (250–500 μs, 30–50 Hz, 30–60 s ON, 10–60 min OFF, and current 1 mA), and they adjusted each patient’s parameters throughout the study. They reported the end parameters had 30 s ON, 20–50 Hz, and 1–2 mA of current; in discussion they wrote that the best results were 250–500 μs pulses, 20 Hz, around 5–10 min of OFF time, and high but tolerable current at 2 mA. Using these parameters, they concluded that the technology was safe, tolerable, and possibly efficacious (Wilder et al., 1991).

It is important to note that those papers so far mentioned have an important caveat. In each paper, patients had “control periods” of no stimulation. As Wilder et al. (1991) noted, there was an apparent cumulative long-term effect of treatment, so how valid could those control periods truly be? Though these were not parametric studies, the numbers used here are largely the methodological foundation of future work.

Ben-Menachem (1994 and 1999) followed up on this research with a randomized, controlled, double-blind study for partial seizures. Instead of comparing patients to their own control periods, they compared high and low stimulation. “High” stimulation, meaning parameters previously thought to be effective, was compared to “low,” or ineffective. What they published as “typical” high stimulation were 500 μs, 30 Hz, 30 s ON 5 min OFF, 1.5 mA current, and over a total duration of 14 weeks. By comparison, they set typical low stimulation at 130 μs, 1 Hz, 30 s/90 min, and 1.25 mA. In summary, the control group had shorter pulses, less current, lower frequency, and longer OFF periods. Their results showed that “high” VNS was tolerable and effective (Ben-Menachem et al., 1994a, 1999). George et al. (1995) and Handforth et al. (1998) used similar parameters against a “low” active control to study partial seizures (George et al., 1995; Handforth et al., 1998). A meta-analysis confirmed that the canonical “high” stimulation had an effect on >50 and >75% decreases in seizure frequency (Ghani et al., 2015).

DeGiorgio et al. (2001) completed a retrospective study of a long-term VNS trial. They analyzed each of the main parameters (pulse width, current, frequency, and ON/OFF time) in patients over 12 months. Though the trial had active and control, the clinicians could adjust the parameters every few months within the range approved for FDA treatment. The analysis found that there may have been some correlation between lower OFF times and response rate and seizure frequency; they argued that the data shows a beneficial effect of lowering OFF-time for those who are initially resistant to treatment. However, more importantly, they did not find any statistically significant association between any other parameter and treatment effect (DeGiorgio et al., 2001).

In a later paper they noted that many of the parameters had a history of uncontrolled studies and possible confounds. They designed a study to focus specifically on ON/OFF times as a duty cycle: 7 s/18 s, 30 s/30 s, and 30 s/3 min, which correspond to 28, 50, and 14.3% duty cycles, respectively. They found that all had similar seizure reductions and proportion of patients who responded at least 50%. However, the 30 s/3 min group had the earliest significant response and the highest number of 75% responders, so the authors concluded that it was likely the optimal ON/OFF for the initial 3 months (DeGiorgio et al., 2005).

Epilepsy is a clinical application of VNS that has a strong history and the convergent parameters are outlined in Table 2. There are several other studies included in the convergent parameters, however, not discussed in detail (Marrosu et al., 2003; Siddiqui et al., 2010; Marras et al., 2013; De Taeye et al., 2014; Fraschini et al., 2014; Orosz et al., 2014; Ryvlin et al., 2014; Boon et al., 2015).

**TABLE 2:** Human Epilepsy (Summary parameters of 19 studies).

	**Pulse Width**	**Frequency**	**On/Off time**	**Time administered**	**Current**
Most common Parameter	500 μs (10 uses)	30 Hz (9 uses)	30 s/5 min (7 uses)	No Common	No Common
Range of Parameters	130–500 μs	20 – 50 Hz	7–120 s ON/18 s – 60 min OFF	30 s – 24 mo	0.25–3.75 mA

## VNS for Treatment Resistant Depression (TRD)

Vagus nerve stimulation as a treatment for depression followed FDA-approval for VNS for epilepsy. Much of the early research reported effects in patients who had VNS implants for epilepsy.

Elger et al. (2000) noted positive mood changes in prior VNS epilepsy trials, but with the caveat that it was difficult to identify whether mood changes were due to reduced seizures, improved quality of life, or some other reason. They designed a study that focused on this association within a larger randomized control trial for epilepsy. They measured eight psychiatric rating scales, two of which pertained to depressive mood and symptoms. They showed mood improvements that were independent of seizure improvement. The parameters used were like the “high” paradigm used in epilepsy: 500 μs, 30/300 s, 6 months duration, and maximum tolerability up to 1.75 mA (Elger et al., 2000).

That same year, a multicenter trial for VNS specific to treatment-resistant depression used parameters familiar by now: 500 μs, 20–30 Hz, and 30 s/5 min; the minor differences are that current was increased to a comfortable level, rather than the maximum level tolerable, and treatment lasted 10 weeks. They found that around 40% of subjects showed at least a 50% decrease in Hamilton Rating Scale for Depression (HRSD) scores, with similar results seen in other depression scales used in the secondary analysis (Rush et al., 2000).

Soon after, Bohning et al. (2001) devised a way to simultaneously activate VNS and capture fMRI. They demonstrated BOLD signals in regions associated with vagus afferent effects in several patients. They used a smaller but more rapid duty cycle (7 s ON, 108 s OFF, 6.1% cycle) than Elger et al. (2000) or Rush et al. (2000), but this is understandable given the different aims of the project (Bohning et al., 2001).

Mu et al. (2004) published the major parametric study for VNS as a depression treatment. They measured VNS effect with fMRI markers of depression and varied the pulse width (130, 250, or 500 μs) over three consecutive scans in twelve participants. They concluded that 250 and 500 μs had a greater association than 130 μs for global brain activation, while 130 and 250 μs had an association for global deactivation. The majority of the studies reviewed for the depression segment of this review used 500 μs.

Table 3 shows that many of the VNS for depression papers reviewed for this review share a common pulse width (500 μs) and ON/OFF time (30 s/5 min). There is evidence that VNS may aid depression treatment, but future work remains before widespread clinical use. There are several other studies examined in this section and referred to in the table but not discussed in detail (Sackeim et al., 2001; Lomarev et al., 2002; Mu et al., 2004; Nahas et al., 2005, 2007; Zobel et al., 2005; Conway et al., 2006, 2012; Pardo et al., 2008; Cristancho et al., 2011; Kosel et al., 2011; Aaronson et al., 2013; Hein et al., 2013; Fang et al., 2016; Rong et al., 2016; Perini et al., 2017; Tu et al., 2018).

**TABLE 3:** Human Depression (Summary parameters of 20 studies).

	**Pulse Width**	**Frequency**	**On/Off time**	**Time administered**	**Current**
Most common Parameter	500 μs (12 uses)	20 Hz (13 uses)	30 s/5 min (9 uses)	6 mo (4 uses)	No Common
Range of Parameters	130–500 μs	1.5–30 Hz	7 s – 30 min ON/41–600 s OFF	14 min – 12 mo	0.13–6 mA

## Facilitating Neuroplasticity With VNS

One area of research that has grown rapidly in the past few years has examined the relationship of vagus afferents and neuroplasticity. Many of these studies look at different kinds of injury repair, motor learning, memory, and hearing, and while this is not an all-inclusive list, we can assume that demonstrating plasticity in any of these domains has some generalizability to the others. We primarily will focus on VNS-paired behavioral interventions that rapidly accelerate learning, reorganize cortical networks, and facilitate recovery post-brain injury.

### VNS-Paired Plasticity

Engineer et al. (2011) first paired VNS with tones and demonstrated that they could make targeted changes in A1 as measured by microelectrode mapping. They investigated whether VNS might have some use in tinnitus treatment. If over-represented frequencies can cause the disease, then increasing cortical representation of non-tinnitus tones may correct that imbalance. VNS paired with multiple tones had significant effects in behavioral testing and A1 responses in rat models (Engineer et al., 2011).

Another study used the same parameters but examined the rates of tone trains. Assuming from literature that rat A1 neurons typically respond to tones around 10 pulses per second, they paired VNS with more or less rapid trains. They showed that rapid pairing increased neuronal ability to follow rapid trains, while slow pairing decreased their ability to follow rapid trains (Shetake et al., 2012). When researchers paired rat VNS to speech sounds, A1 response increased to those sounds and not to novel speech sounds. The same parameters were used as the previous study (Engineer et al., 2015).

Pena et al. (2013) is one of many rat studies that have paired VNS to audio tones. Others would use tones as the stimulus alone instead of as conditioning. Researchers then examined the primary auditory cortex (A1) afterwards as a measurement of plasticity. Again, assuming that plasticity is a widespread underlying mechanism of VNS effects, findings in A1 are not in total isolation from findings in primary motor or somatosensory cortices. Many of them share VNS parameters (100 μs, 30 Hz, 500 m s train, and 0.8 mA), so there is a lot more comparability between these papers. An important concept to keep in mind for this section is the idea of tonotopy, or tone-mapping, in the auditory; peak response in areas of auditory cortex correspond to regions of the frequency spectrum.

Borland et al. (2016) investigated the question of whether current intensity affects VNS-paired plasticity. In their study, only current intensity was varied: they assigned rats to 0.4, 0.8, 1.2, or 1.6 mA for VNS paired with a given tone. After 20 days of paired stimulation they measured the area of A1 responsive to frequencies near the paired tone. 0.4 and 0.8 mA rats had significantly different area-response changes compared to control (naïve) rats, whereas the higher intensities failed to reach significance (Borland et al., 2016). This largely supports the effective level of current found in other studies, although it cannot directly support the inverted-U pattern.

The next parametric study used the same frequency, train length, and duration as the previous studies, but varied pulse width (100 or 500 μs) and current intensity (0.2 or 0.8 mA). They built on previous research that had repeatedly shown an inverted-U pattern for current intensity, as well as the levels of each that drove plasticity (100 μs and 0.8 mA). However, they designed this study to examine the relationship of pulse width and current. Starting from the customary parameters, dropping the current to 0.2 mA abolished the VNS benefits to plasticity. However, low current intensity (0.2 mA) with extended pulse width (500 μs) still had an effect, albeit less than the customary parameters. This suggests that there is interaction between these parameters. Furthermore, taken together with other research, they argued that shorter pulse width may have a permissive effect on current, in the sense that it allowed a wider range of currents to drive plasticity (Loerwald et al., 2018).

Recent research has also taken a closer look at the influence of timing on VNS and A1. Researchers varied the number of VNS-tone pairings and the amount of time that elapsed between them in acoustic trauma rat models. Inter-stimulus intervals correspond to OFF times; they found that shortened intervals (8 s instead of the standard 30 s) drove plasticity less than the standard protocol, while longer intervals (120 s) drove plasticity roughly as much as standard. Reducing the number of pairings (from the standard 300 pairings to only 50) abolished the plastic effects (Borland et al., 2018).

### Functional Motor Improvements in Animals

Behaviorally, VNS paired rehabilitation showed produced functional improvement in motor tasks in rats with TBI. Researchers speculated about a “Norepinephrine hypothesis” so far suggested in VNS for epilepsy and memory might also apply to motor recovery. They used 500 μs pulses, 20 Hz frequency, 30 s ON, and 29.5 min OFF with current 0.5 mA and duration 14 days. Though the behavioral results were significant, they found no histochemical differences. They also argued that their results supported the idea of plasticity in functional recovery (Smith et al., 2005).

Rats trained in a specific movement and paired it with VNS for 5 days showed a greater area of motor cortex responding to the paired (Porter et al., 2012). There are two important differences between these findings and Smith et al. (2005). First, whereas the previous paper used general motor tasks in injured rats, this paper focused on a specific movement in healthy ones. Second, Porter et al. (2012) used very different parameters because they cited them from Engineer et al. (2011) – an A1 plasticity paper (100 μs, 30 Hz, 500 m s train, and 0.8 mA). They argued that the mechanisms of plasticity may be similar in different areas of the brain, so a motor pairing should operate in the same way (Porter et al., 2012).

When researchers induced motor cortex ischemia in rats that they previously trained to a task, rats paired with VNS post-ischemia showed twice as much improvement compared to control. They cited Porter et al. (2012) and Engineer et al. (2011) for their parameters, though their treatment duration was longer than in either – 100 μs, 30 Hz, 500 m s train, 25 day, and 0.8 mA (Khodaparast et al., 2013).

Two studies by Seth Hays in 2014 (Hays et al., 2014a,b) used 30 Hz and 0.8 mA of current, similar to preceding animal studies in epilepsy. The timing of stimulation pulses was different on a few scales. The pulse width used was shorter (100 μs); this width is common in many rehabilitation and general plasticity experiments. Furthermore, the ON period was 500 ms triggered by movement, in contrast to the usual 30 s ON and regular OFF periods seen in epileptic studies. The irregular OFF period is because of a concept of paired timing in rehabilitation VNS and plasticity that VNS is effective in plasticity only when given in a very small window of time near the target function. Both studies demonstrated a significant improvement with VNS-paired rehabilitation and further confirm the importance of time-pairing the stimulus to action. They later confirmed this finding in aged rats (Hays et al., 2016). In rats with ischemic lesions, VNS not only augmented rehabilitation, but the effects lasted months after treatment ended, and carried some generalizable improvement to untrained tasks (Meyers et al., 2018). Impaired signaling of norepinephrine, serotonin, or acetylcholine can prevent the efficacy of VNS rehabilitation (Hulsey et al., 2019; Meyers et al., 2019). VNS also improved rehabilitation in cervical spine injury rats (Darrow et al., 2020b). There was benefit to somatosensory rehabilitation using similar parameters to those used by motor recovery experiments (Darrow et al., 2020a).

### Implanted VNS for Adult Stroke Rehabilitation

The final portion of this section covers the limited research of VNS-paired rehabilitation in human subjects. A published protocol for a randomized crossover prospective clinical trial to find the effect of VNS-pairing in human subjects with traumatic brain injury (TBI) used the parametric ranges 10–30 Hz frequency and 0.5–2.5 mA current. Their pulse width and ON/OFF time resembled human VNS for epilepsy – 500 μs pulses for 30 s/5 min, respectively (Shi et al., 2013). However, contacting the senior author, it seems that the arrival of Hurricane Sandy prevented any follow-up on this paper.

A pilot randomized control trial studied VNS-paired rehabilitation in humans with ischemic stroke deficits in a pilot randomized control trial. 9 received VNS pairing and 11 received standard rehabilitation. Unlike the ranges used in Shi et al. (2013), the parameters they used over 6 weeks were identical to those used in rat plasticity research (100 μs pulse, 30 Hz, 500 m s train, and 0.8 mA). They found a significant improvement in upper extremity performance scores when analyzing the data per protocol, but not when analyzing it as intention-to-treat. It seems only one patient was lost from the control group between these two analyses for taking a medication that met exclusion criteria (Dawson et al., 2016).

An important case study on somatosensory rehabilitation in humans paired 5 weeks of the standard VNS plasticity parameters (100 μs, 30 Hz, 500 m s train, and 0.8 mA) with sensory training in a single human subject with deficits in the left arm. The subject improved over time in several measures of tactile sense. Though uncontrolled, it is worth noting that the stroke that caused the patient’s symptoms happened 2 years previous, so it is difficult to imagine this recovery was spontaneous (Kilgard et al., 2018).

The next step was to compare VNS to a sham-VNS control. Researchers implanted VNS in 17 subjects (8 active, 9 sham) with upper extremity deficits following ischemic stroke. They showed that VNS-paired therapy patients had significantly more responders according to Fugl-Meyer Assessment – Upper Extremity (FMA-UE) scores, as well as significant long-term improvements in Wolf Motor Function tests. However, it is worth noting that several other motor assessments failed to show significant differences between the groups. The design of the rehabilitation is also important: each subject had a period of in-clinic therapy and at-home therapy, both of which delivered 500 ms of VNS at the standard 100 μs pulses, 30 Hz, and 0.8 mA. In the former (6 weeks), a therapist could assess the exercise and deliver VNS timed to each successful movement (500 ms train); in the latter (60 days), subjects were given a 30-min daily exercise regimen to do at home, at the start of which they would use a magnet to turn on the VNS for 30 min (500 ms ON every 10 s). There were not significant differences between group FMA-UE scores at the end of in-clinic therapy (Kimberley et al., 2018). Other studies in this section have highlighted the importance of timing in pairing, so it is possible that this design had some influence on the results. Recently, Dawson et al. (2021) completed the largest implanted VNS trial for motor rehabilitation that reliability demonstrates the efficacy of cervically implanted VNS to improve motor function when paired with post-stroke motor rehabilitation.

In conclusion, the field of VNS in plasticity may be one of the younger sub-fields, but parametrically it is one of the most consistent. In addition, it has studies optimizing almost every parameter.

Plasticity is a consistent and strong field of VNS research that may shed light on many fundamental principles of neuroscience as a whole. There are several other studies examined in this section and Table 4 but not discussed in detail (Clark et al., 1995, 1999; Bajbouj et al., 2007; Biggio et al., 2009; Vanneste et al., 2017; Buell et al., 2019; Hulsey et al., 2019; Meyers et al., 2019; Sanders et al., 2019; Darrow et al., 2020a,b).

**TABLE 4:** Neuroplasticity and Rehabilitation (Summary parameters of 33 studies).

	**Pulse Width**	**Frequency**	**On/Off time**	**Time administered**	**Current**
Most common Parameter	100 μs (26 uses)	30 Hz (26 uses)	500 ms train (24 uses)	20 d and 6 w (6 uses)	0.8 mA (25 uses)
Range of Parameters	100–500 μs	7.5–120 Hz	500 ms – 30 s ON/29.5 s – 29.5 min OFF	30 s – 18 mo	0.2–3.2 mA

## Transcutaneous Auricular Vagus Nerve Stimulation (taVNS)

This new non-invasive form of VNS should consider the century of VNS literature to guide its administration. A note on literature conventions, however, it is important to note current is applied to the skin, rather than directly to the nerve. Here we will use taVNS to refer to all transcutaneous VNS acting on the ear (Badran et al., 2019; Farmer et al., 2020). This section is not intended to be an exhaustive review of all taVNS applications, however, we have chosen a representative pool of work from the rapidly growing field of neurological and psychiatric taVNS applications (Wang et al., 2021).

### taVNS Human Parametric Studies

Ventureyra proposed the method underlying taVNS in 2000, combining the concepts of transcutaneous electrical stimulation of the nervous system (TENS), the anatomy of ear innervation, and research in acupuncture (Ventureyra, 2000). Later researchers applied this idea by running current through electrodes on several locations on the ear and measured significant vagus sensory evoked potentials from stimulation of the tragus. VSEP is measured from the scalp, so they could conclude that stimulation had an effect, but not exactly where or what (Fallgatter et al., 2003). So, while these results were promising, more work remained to determine whether this stimulation targeted areas associated with vagal afferents.

To our knowledge, the first parametrically relevant study stimulated the ear and recorded BOLD changes in fMRI, as well as pre- and post-psychometric assessments. As this was unbroken ground, they first ran a test series of several people to find the optimal stimulation parameters; however, they merely wrote that these were based on “ratings of quality of subjective perception,” so it is unclear how rigorously they optimized the levels. Current intensity was set at perceptual threshold and just under pain threshold. They used 20 μs pulses, 8 Hz frequency, and ON/OFF time of 30 s/2 min (for psychometric tests) or 30 s/1 min (fMRI). Compared to sham, they found BOLD patterns like those seen in conventional VNS – decreased BOLD in limbic areas, increased BOLD in the thalamus, insula, and precentral gyrus. Psychometric scores showed significant subjective improvement of well-being in the taVNS group, whereas sham subjects saw worsening of subjective feelings (Kraus et al., 2007). Another fMRI study by this group validated these results using 20 μs, 8 Hz, 30 s/1 min cycles, and current just below pain threshold. In addition, they stimulated the anterior and posterior ear canal separately. Anterior and posterior stimulation both increased BOLD in the insula, but work in opposition in other areas; anterior canal stimulation decreased BOLD in the parahippocampus, posterior cingulate, and thalamus, while increasing BOLD in the locus coeruleus and solitary tract (Kraus et al., 2013). Lastly, a 2018 fMRI study further demonstrated the positive neurophysiological effects of supra-threshold taVNS delivered 500 μs, 25 Hz, in 30 s blocks when compared to sham using concurrent taVNS/fMRI (Badran et al., 2018b).

The taVNS field is still in its infancy, however, the literature thus far illustrates a diversity of other considerations in the parameters used. Current intensity is typically administered between perceptual and pain threshold – a dosing metric to control for pain as a confound. frequency often hovers between 20 and 30 Hz, but the other parameters vary without noticeable pattern. Badran et al. (2018) conducted a series of experiments that aimed to optimize taVNS using cardiac biomarkers. In back-to-back studies, they investigating varying pulse width and frequency while keeping current intensity standardized at 2 × perceptual threshold (Badran et al., 2018d). taVNS was administered during 1 h sessions, with ON/OFF 60 s/270 s (trial 1) or 60/150 s (trial 2). They varied frequency (1, 10, and 25 Hz) and pulse width (100, 200, and 500 μs) in nine combinations in the first trial, with a second trial using only the two best combinations from trial 1. They used heart rate change to measure the strength of vagus activation. Their results showed that 500 μs and 10 Hz had the strongest effect on heart rate, while 500 μs 25 Hz had the next strongest effect. Recall that most taVNS papers use 20–30 Hz frequency; while some have used 500 μs, it is far from a majority. They note that heart rate is an indirect way to assess the central effects of taVNS, so replication of these trials in imaging are needed in the future (Badran et al., 2018d). It will also have to be validated for different disciplines – for example, other research has found that 1 Hz was significantly better than 25 Hz at reducing headache frequency in chronic migraine patients (Straube et al., 2015).

Auricular neurostimulation introduces non-neural tissue between the electrodes and the nerve – which acts as an insulator and allows for further variation in parameters to be explored, including higher frequencies and intensities that may not be otherwise safely administered in animals without causing a lesion in the nerve. Without a consensus on ideal parameters, taVNS researchers carried on to human clinical trials, often using parameters similar to those administered in cervically implanted VNS analogs.

### taVNS Human Clinical Trials

Following these functional imaging studies, taVNS began to emerge for a variety of different applications with widely divergent parameters. 2012 saw several pilot studies evaluating the feasibility of taVNS in different disease treatments. A single-armed pilot study applied taVNS for 3–10 weeks in patients with chronic tinnitus. They measured clinical electrocardiograms in clinical exams every few weeks. They found that taVNS was associated with possible QRS shortening. There were two adverse events, but the authors concluded that it was likely not due to stimulation. The researchers set taVNS parameters at 25 Hz, 30 s ON, 180 s OFF, and current between perceptual and pain threshold (approximate range 0.1–10 mA) (Kreuzer et al., 2012). Adverse events caused an early termination of the first phase, so they followed up with a second phase using a different stimulating device, 30 s ON/30 s OFF, and two fewer hours of stimulation per day. Altogether, the Kreuzer tinnitus work concluded safety, feasibility, significant changes from baseline for some clinical scores, but no decrease in clinical complaints (Kreuzer et al., 2014).

Other studies investigated the effect of taVNS in patients with resistant epilepsy. They applied taVNS for an hour three times daily for 9 months, and then recorded a week of video-EEG. Patients kept seizure diaries. Parameters used were 300 μs pulses, 10 Hz, 1 h ON, and current as high as the patients could tolerate regularly. They concluded that taVNS was safe and tolerable for long treatment courses, and five of the seven patients that completed the trial saw fewer seizures. However, the caveat to that tolerability is that three of the original ten subjects dropped out because the protocol was too much for them to do day-to-day, or for technical problems, or due to direct side effects (Stefan et al., 2012).

A full double-blind randomized clinical trial for taVNS in resistant epilepsy used different stimulation parameters: 250 μs, 25 Hz (or 1 Hz for the active control), ON/OFF 30 s/30 s, 20 weeks of treatment, and current set between perceptual and pain thresholds (average 1.02 mA control or 0.50 mA treated, with a statistically significant difference between the two). They showed that the treatment group that completed the treatment had a significant decrease in seizure frequency not seen in the control, but both groups had similar responder rates. They were unable to conclude that the 25 Hz was superior to the control (Bauer et al., 2016).

Two specific subsets of taVNS called Respiratory-gated Auricular Vagal Afferent Nerve Stimulation (RAVANS) (Garcia et al., 2017) and Motor Activated Auricular Vagus Nerve Stimulation (MAAVNS) (Cook et al., 2020a) emerged as closed loop solutions to the parametric problem. RAVANS works by the idea that inhalation induces transient inhibition of vagus nerve activity. Investigators have applied RAVANS to chronic pain subjects. The “ON” period is a train of 500 ms in response to exhalation, while the “OFF” period lasts until the start of the next expiration. They designed a counterbalanced crossover study for taVNS in patients with chronic pain in the pelvis and tested each patient with RAVANS or sham stimulation at least a week apart. Parameters were 450 μs pulses, 30 Hz, 30-min treatment sessions, and current set just below pain threshold. RAVANS has not only shown promise in treating pain disorders, but also other neurological disorders like migraine (Garcia et al., 2017). These studies suggest that taVNS effects are likely compounded by the respiration-induced vagal effects at the brain stem. MAAVNS, however, pairs taVNS with motor activity, using 500 μs pulses at 25 Hz that are turned on during the duration of a targeted motor activity (Cook et al., 2020b). MAAVNS has been demonstrated to be a promising neurorehabiltiation tool (Badran et al., 2018c, 2020) and in early studies has demonstrated promise in facilitating motor learning in neonates MAAVNS is further continued to be explored in adult post-stroke rehabilitation trials.

Further exploration of open-loop taVNS for pain control used forty-eight healthy subjects in a taVNS/sham crossover control. Their stimulation used 250 μs pulses at 25 Hz, 1 h ON, and current intensity between perceptual and pain thresholds (reported 0.25–10 mA). They cited Vonck et al. (1999), a study of conventional VNS in epilepsy, for the frequency. Their results showed some analgesic effects for mechanical pain and noxious heat (Busch et al., 2013).

Building upon all the promising animal and human implanted VNS work that has come out of Texas by groups led by Hays, Kilgard, and Engineer, many researchers have pushed taVNS into the motor rehabilitation space. Redgrave et al. (2018) conducted an open label pilot study using taVNS concurrently with post-stroke upper limb rehabilitation in 18 1-h sessions (25 Hz, 100 μs pulse width) with promising improvements in motor function. Baig et al. (2019) explored a similar post-stroke intervention as Redgrave, and demonstrated promising sensory recovery effects. Unlike Redgrave and Baig who used therapists to conduct the rehabilitation training, Capone et al. (2017) utilized robots to create a taVNS-paired robotic intervention for post-stroke rehabilitation. Lastly, the closed-loop, intelligent, MAAVNS system that has shown early success in neonates has been translated to adult upper limb rehabilitation and is being investigated in a small randomized trial (ClinicalTrials.gov Identifier: NCT04129242). This MAAVNS system delivers taVNS in a temporally specific fashion that builds upon the animal work described earlier in this manuscript.

In conclusion, ongoing work in taVNS may radically change the field and eliminate the barrier of surgery to many patient populations. It is important to understand that aside from parametric considerations, taVNS is sensitive to stimulation target that, although is not discussed in this review (Badran et al., 2018). There are several other studies examined in this section and Table 5 but not discussed in detail (Hein et al., 2013; Clancy et al., 2014; Capone et al., 2015; Frangos et al., 2015; Hasan et al., 2015; Fang et al., 2016; Rong et al., 2016; Yakunina et al., 2017; Badran et al., 2018b; Tu et al., 2018).

**TABLE 5:** taVNS (Summary parameters of 22 studies).

	**Pulse Width**	**Frequency**	**On/Off time**	**Time administered**	**Current**
Most common Parameter	250 μs (5 uses)	25 Hz (12 uses)	30 s ON (9 uses but variable OFF)	No Common	Supra-Threshold (10 uses)
Range of Parameters	20–500 μs	1–30 Hz	0.5 s – 30 min ON/30–270 s OFF	6 min – 9 mo	0.13–50 mA

## Summary and Conclusion

Vagus nerve stimulation is an important brain stimulation modality that has a history spanning over 150 years. Fascinatingly, there is still no consensus parameter that is the “best” parameter for VNS. There is likely no perfect combination of current intensity, pulse width, frequency, duty cycle, and duration - the more likely case is that there is a wide range of parameters that are biologically active and induce promising behavioral effects. Furthermore, there is an abundance of promising work that future research will uncover about the current-pulse width relationship in the plasticity field.

This manuscript is intended to serve as a historical perspective and guide future VNS trials and research. There are three key take home messages from this manuscript that we have synthesized below:

(a) **Current intensity and pulse width are critical -** From much of the work described in this manuscript, increasing current intensity gradually increases release of neurotransmitters like norepinephrine (Roosevelt et al., 2006; Follesa et al., 2007) and increasing firing rate of cells in the locus coeruleus (Hulsey et al., 2017). Many applications of implanted VNS titrate the intensity to comfort, and nearly all taVNS studies employ supra-threshold stimulation intensity.
The vagus nerve is a bundle of thousands of nerves, each with their own activation thresholds. The majority of these ascending fibers are small, unmyelinated C fibers, whereas the remaining are myelinated A and B fibers. A-beta fibers have the lowest firing threshold, which would be activated first, but not until higher current intensities are C fibers activated (Collins et al., 1960). The fundamentals of nerve conductance and firing thresholds should be considered in VNS, however, when directly stimulating the nerve, discomfort may impede the increasing of the intensity. Furthermore, the current intensity and pulse width interaction should be considered. When current intensities are equal, increasing pulse width allows for increased VNS effects (Loerwald et al., 2018). However, achieving higher current intensities may be only tolerable at lower pulse widths. This interaction needs to be further explored.
(b) **Frequency seems to need less precision –** In the review of these over 100 studies, it seems that the range of frequencies that have been carried onward over the years. Most manuscripts seem to settle on a frequency between 20–30 Hz, which has been shown to be more biologically active in both in implanted functional neuroimaging as well as in taVNS optimization trials. There has yet to be a broad parametric search for optimal frequency, however, the current state of the research suggests many of the behavioral effects are found in the range of the original anti-epileptic parameters of the early 1990’s (Ben-Menachem et al., 1994b). There is a need to explore the systematic testing of varying frequency.
(c) **On/Off times may be more state dependent than previously believed –** much of the work described here explores a wide range of On/Off times, and mostly were employed early in VNS development to avoid lesions to the nerve and as a means to save battery life in the implant. The early work settled on 30 s ON, 5 min OFF, and not much has changed in the implant space. As we move to neuroplastic effects, ON/OFF times are less critical, and temporal pairing of stimulation bursts with behavioral interventions was more effective (Hays et al., 2014a). As we move to taVNS, safety and power issues of the implanted VNS have been resolved as external pulse generators can be easily recharged and stimulation is not delivered directly to the nerve. Pairing of taVNS with behaviors is also emerging as shown in both the RAVANS (Garcia et al., 2017) and MAAVNS (Cook et al., 2020b) applications.

As VNS research grows, we should consider the historical perspective and further optimize the parameter space. There is room for improvement and a large body of literature that can be improved upon as VNS continues to emerge as a promising neuromodulation modality.

## Author Contributions

All authors listed have made a substantial, direct and intellectual contribution to the work, and approved it for publication.

## Conflict of Interest

BB is listed as inventor on pending or issued patents in the brain stimulation field, assigned to Bodhi NeuroTech, Inc., and the Medical University of South Carolina. BB serves as a consultant to companies developing non-invasive vagus nerve stimulation. The remaining authors declare that the research was conducted in the absence of any commercial or financial relationships that could be construed as a potential conflict of interest.
